# Analysis of bZIP Transcription Factor Family and Their Expressions under Salt Stress in *Chlamydomonas reinhardtii*

**DOI:** 10.3390/ijms19092800

**Published:** 2018-09-17

**Authors:** Chunli Ji, Xue Mao, Jingyun Hao, Xiaodan Wang, Jinai Xue, Hongli Cui, Runzhi Li

**Affiliations:** Institute of Molecular Agriculture and Bioenergy, Shanxi Agricultural University, Taigu 030801, China; jichunnli@sxau.edu.cn (C.J.); maoxue@sxau.edu.cn (X.M.); haojingyun@stu.sxau.edu.cn (J.H.); wangxiaodan@sxau.edu.cn (X.W.); xuejinai@sxau.edu.cn (J.X.); cuihongli@sxau.edu.cn (H.C.)

**Keywords:** *Chlamydomonas reinhardtii*, bZIP transcription factors, salt stress, transcriptional regulation, photosynthesis, lipid accumulation

## Abstract

The basic leucine-region zipper (bZIP) transcription factors (TFs) act as crucial regulators in various biological processes and stress responses in plants. Currently, bZIP family members and their functions remain elusive in the green unicellular algae *Chlamydomonas reinhardtii*, an important model organism for molecular investigation with genetic engineering aimed at increasing lipid yields for better biodiesel production. In this study, a total of 17 *C. reinhardtii* bZIP (CrebZIP) TFs containing typical bZIP structure were identified by a genome-wide analysis. Analysis of the CrebZIP protein physicochemical properties, phylogenetic tree, conserved domain, and secondary structure were conducted. *CrebZIP* gene structures and their chromosomal assignment were also analyzed. Physiological and photosynthetic characteristics of *C. reinhardtii* under salt stress were exhibited as lower cell growth and weaker photosynthesis, but increased lipid accumulation. Meanwhile, the expression profiles of six *CrebZIP* genes were induced to change significantly during salt stress, indicating that certain CrebZIPs may play important roles in mediating photosynthesis and lipid accumulation of microalgae in response to stresses. The present work provided a valuable foundation for functional dissection of CrebZIPs, benefiting the development of better strategies to engineer the regulatory network in microalgae for enhancing biofuel and biomass production.

## 1. Introduction

Microalgae are considered to be one of the most promising feedstocks for renewable biofuel production. However, the shortage of inexpensive algal biomass currently hampers microalgae-based biofuel industrialization [1]. Microalgae accumulate high level of lipids, mainly in the form of triacylglycerol (TAG), when subjected to nutrient deprivation and other stresses [2,3,4,5]. In parallel, these adverse conditions also limit algal biomass accumulation. Consequently, genetic engineering to achieve an optimized balance between oil accumulation and biomass growth may represent an effective strategy for the improvement of microalgae biofuel yield. Therefore, it is necessary to comprehensively analyze the underlying molecular mechanisms that mediate stress-induced accumulation of oil in microalgae, particularly to identify the key transcription factors (TFs). The unicellular algae *Chlamydomonas reinhardtii* is the de facto model organism for research in microalgae. Various types of omics data for *C. reinhardtii* are available, including its full genome [6], the proteomics and metabolomics analysis, and the phenotype transition during N starvation [7,8,9,10,11,12,13]. These achievements provide the basis for further investigation into oil metabolism and regulation in *C. reinhardtii*, which would shed light on the development of rational strategies for sustainable production of microalgae biofuel.

Transcription factor (TF) encoding genes are considered as contributing to the diversity and evolution in plants. Identification of the transcriptional factors and their cognate transcriptional factor binding-sites is essential in manipulating the regulatory network for desired traits of the target molecules [14]. Moreover, the control of transcription initiation rates by transcription factors is an important means to modulate gene expression, and then regulate the organism growth and development [15]. The basic region-leucine zipper (bZIP) family is one of the most conserved and wildly distributed TFs present in multiple eukaryotes. To date, they have been extensively investigated in many plants including *Arabidopsis*, rice, tomato, maize, sorghum, carrot, and so forth [16,17,18,19,20,21,22]. The bZIP TFs have been found to mediate various biological processes, such as cell elongation [23], organ and tissue differentiation [24,25,26], energy metabolism [27], embryogenesis and seed maturation [28], and so forth. The bZIP TFs also participate in plant responses to biotic and abiotic stresses, including pathogen defense [29,30], hormone and sugar signaling [31,32], light response [33,34], salt and drought tolerance [20,35], and so forth. Typically, bZIP TFs contain a conserved 40–80 amino acid (aa) domain which has two structure motifs: A DNA-binding basic region and a leucine zipper dimerization domain [15]. The basic region composing of around 20 amino acid residues with an invariant N-X7-R/K-X9 motif is highly conserved, and the main function of this region is for nuclear localization and DNA binding. The leucine zipper containing a heptad repeat of leucine is less conserved, with the property for recognition and dimerization [21]. The diversified leucine zipper region is located exactly 9 aa downstream from the C-terminal of the basic region. Although bZIP family members were intensively reported to mediate diverse stress responses in higher plants, little attention has been paid to studying bZIP TFs and their downstream target genes on a genome-wide scale in microalgae.

A total of 147 putative TFs of 29 different protein families have been identified in *C. reinhardtii*, including 1 WRKY, 4 bHLH, 5 C2H2, 11 MYB, 2 MADS, 7 bZIP TFs, and so forth [36]. However, functions remain unclear for the majority of these TFs. The bZIP family is also one of the four largest TF families in oleaginous microalgae *Nannochloropsis* [14], showing that some bZIPs were putatively related with the transcriptional regulation of TAG biosynthesis pathways in *Nannochloropsis.* Therefore, the present study focused on the genome-wide identification of bZIP TFs in *C. reinhardtii* and their functional analysis, with an objective to elucidate the mechanism underlying the regulation of fatty acid and oil accumulation, and photosynthesis in microalgae, particularly under stresses.

In this study, the bZIP sequences of *C. reinhardtii* were intensively identified using a proteomic database, and a total of 17 CrebZIP TFs were obtained after removing the redundancy. Bioinformatics tools were employed to perform a detailed analysis of their genetic structure, chromosome distribution, classification, protein domain, and motifs, as well as evolutionary relationship. Furthermore, the physiological and photosynthetic characteristics of *C. reinhardtii* under salt stress were measured, including biomass concentration, lipid and pigment contents, as well as chlorophyll fluorescence variation. Finally, to infer the potential functions of these CrebZIPs, the expression profiles of *CrebZIP* genes under salt stress were quantitatively examined using quantitative real time (qRT)-PCR. Thus, these integrated data would provide new insights into comprehensive understanding of the stress-adaptive mechanisms and oil accumulation mediated by bZIP TFs in *C. reinhardtii* and other microalgae.

## 2. Results and Discussion

### 2.1. Identification of C. reinhardtii bZIP Family Members

To perform genome-wide identification of bZIP proteins in *C. reinhardtii*, BLAST and the Hidden Markov Model (HMM) profiles of the bZIP domain were used to screen the *C. reinhardtii* genome and proteome database, with bZIP sequences from *Arabidopsis* as the query. A total of 17 *CrebZIP* genes in *C. reinhardtii* were identified and denominated as *CrebZIP1–CrebZIP17* based on their locations in the chromosome (Table 1).

The number of CrebZIPs obtained here was not consistent with previous reports. Corrêa et al. and Riano-Pachon et al. identified 7 putative CrebZIP TF coding sequences [15,36]. However, study on the evolution of bZIP family TFs among different plants by Que et al. detected 19 bZIP TFs in *C. reinhardtii*. They summarized that the number of bZIP TFs in algae (less than 20) and land plants (greater than 25) differed remarkably, and bZIP TFs might thus have expanded many times during plant evolution [22]. Such difference in bZIP numbers in *C. reinhardtii* might have resulted from different versions of the *C. reinhardtii* genome and protein database, and criteria used in those reports.

Conserved Domain Database (CDD) and Simple Modular Architecture Research Tool (SMART) analysis indicated that the 17 CrebZIP proteins all had typical bZIP conserved domains. Table 1 summarizes their physicochemical properties, including the protein length which ranged from 334 (CrebZIP13) to 2018 (CrebZIP1) amino acids, the corresponding molecular weight which varied from 3,4514.92 to 198,080.64 Da, and theoretical isoelectric point (pI) which varied from 4.96 (CrebZIP13) to 9.55 (CrebZIP12). The great difference in these properties may reflect their functional diversity in *C. reinhardtii*. The minus hydrophility of all CrebZIP proteins and their higher instability index (>40) showed that they were hydrophilic and unstable.

To get the protein structure information of these CrebZIP members, the secondary structure of the proteins was predicted by the PBIL LYON-GERLAND database. The secondary structure information is listed in Table 2, including α-helix, extended strand, and random coil. Of them, random coil accounted for a higher percentage (45.23–72.75%), while extended strand had the lowest proportion (0.42–8.73%). No β-bridge was detected in CrebZIPs.

### 2.2. Phylogenetic and Motif Analysis of CrebZIP Proteins

**Figure 1:**
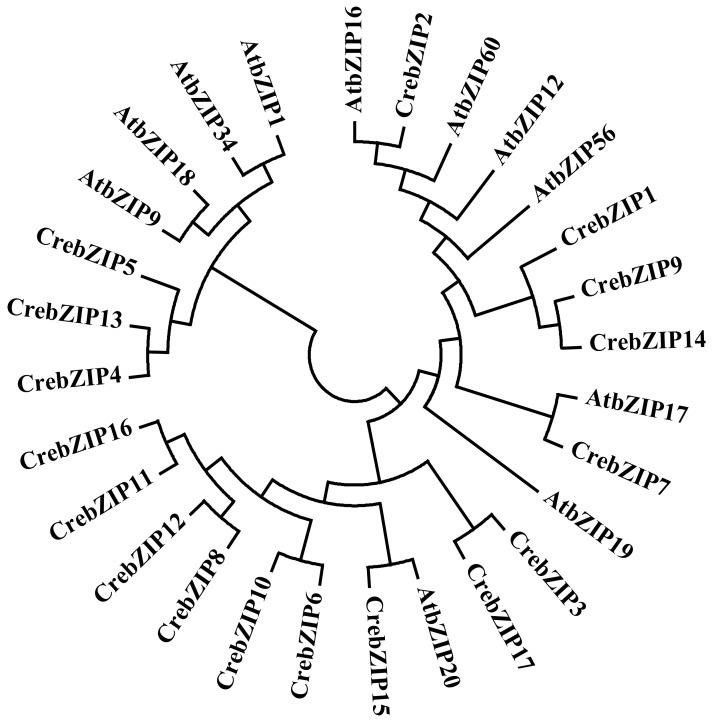
Phylogenetic analysis of *C. reinhardtii* and *Arabidopsis* basic leucine-region zipper (bZIP) proteins. ClustalW was employed to align the protein sequences of 17 CrebZIPs and 11 AtbZIPs representing subgroups A to I, S, and U in *Arabidopsis*. The phylogenetic tree was constructed using the neighbor-joining (NJ) method with MEGA7.0 software. The evolutionary distances were computed using the Poisson correction method with the number of amino acid substitutions per site as the unit. All positions containing gaps and missing data were excluded.

To explore the evolution and classification of CrebZIP TFs, we performed a phylogenetic analysis (Figure 1) of 17 CrebZIP and 11 AtbZIP protein sequences. AtbZIPs were selected according to the classification of *Arabidopsis* bZIP proteins. Ten groups of AtbZIP proteins named Group A, B, C, D, E, F, G, H, I, and S were defined according to the sequence similarity of the basic region and other conserved motifs [17]. Those AtbZIP proteins that did not fit into any group mentioned above were classified as Group U (unknown). One AtbZIP protein was selected from each group respectively, including AtbZIP12 (Group A), AtbZIP17 (Group B), AtbZIP9 (Group C), AtbZIP20 (Group D), AtbZIP34 (Group E), AtbZIP19 (Group F), AtbZIP16 (Group G), AtbZIP56 (Group H), AtbZIP18 (Group I), AtbZIP1 (Group S), and AtbZIP60 (Group U). As shown in Figure 1, most bZIP members from the same species tended to cluster together. Only three CrebZIPs (CrebZIP2, 7, and 15) were grouped together with three AtbZIPs (AtbZIP16, 17 and 20), respectively, forming three subfamilies. Analysis on sequence identity and the similarity of bZIP proteins between *C. reinhardtii* and *Arabidopsis* grouped in the same subfamilies, showed low levels of amino acid conservation between the two species. CrebZIP2 and AtbZIP16 had 10.8% identity and 14.4% similarity, while CrebZIP7 and AtbZIP17 exhibited 10.5% identity and 16.9% similarity. The third pair of CrebZIP15 and AtbZIP20 only had 6.6% identity and 11.6% similarity. The remaining 14 CrebZIPs were grouped into 8 subfamilies, with each containing two CrebZIP members except for CrebZIP1 and CrebZIP5, which were classified as two single-member subfamilies. It is possible that the 14 CrebZIPs may have independent ancestral origins different from the AtbZIPs, in consideration of the fact that *C. reinhardtii* is a lower plant, while *Arabidopsis* is a higher plant. To extend the bZIP analysis to larger lineages of green plants, bZIP members from two bryophytes including *Physcomitrella patens* and *Marchantia polymorpha* were also added into the phylogenetic analysis, as bryophytes are considered as one of the earliest diverging distant land-plant lineages. Table S1 shows 43 *PpbZIP* genes from *P. patens* and 14 *MpbZIP* genes from *M. polymorpha* that were identified. SMART analysis indicated that these bZIP proteins all had the typical bZIP conserved domains. The phylogenetic analysis of bZIP proteins from *C. reinhardtii*, *Arabidopsis*, *P. patens*, and *M. polymophra* indicated that most CrebZIP proteins were also not highly homologous with *P. patens* and *M. polymorpha* bZIPs (Figure S1). Two CrebZIPs (CrebZIP 2 and 6) and two MpbZIPs (MpbZIP4 and 12) clustered together, respectively. However, levels of identity and similarity were very low between the CrebZIPs and MpbZIPs grouped in the same subfamilies. The identity and similarity between CrebZIP2 and MpbZIP4 were 14.5% and 19.8%, respectively, while CrebZIP6 and MpbZIP12 only shared 8.6% identity and 13.4% similarity.

In view of the orthologous bZIP proteins playing a similar role, CrebZIP2 may function like AtbZIP16 (Group G), which mainly linked to light-regulated signal transduction and seed maturation. CrebZIP7 was aligned with AtbZIP17 (Group B), however no functional information was available for members of this group. CrebZIP15 was clustered together with AtbZIP20 from Group D. Members of this bZIP group mainly participated in defense against pathogens, and development [17].

**Figure 2:**
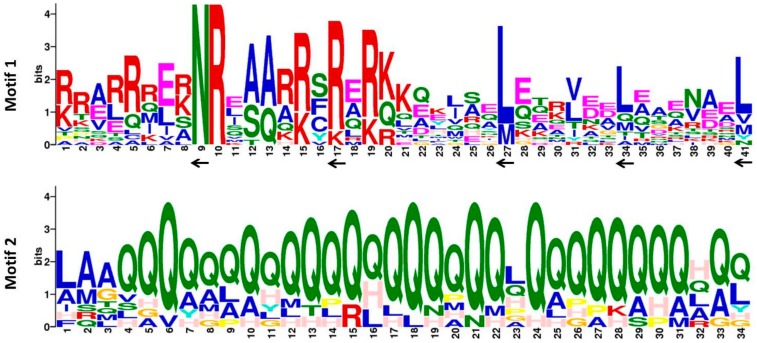
Analysis of the conserved domain in CrebZIP proteins. The conserved motifs in the CrebZIP proteins were identified with MEME software. Conserved sites in the key conserved domain (motif 1) are indicated by “←”.

To obtain insight into the divergence and function of CrebZIP TFs, the conserved motifs in the CrebZIPs were analyzed by MEME software. As depicted in Figure 2, all CrebZIP proteins contained the typical bZIP structure domain (motif 1). In addition, a glutamine (Q) enrichment region (motif 2) was detected in 10 CrebZIP proteins including CrebZIP1, 3, 6, 8, 10, 11, 12, 14, 15, and 16. In general, the basic region of bZIP protein has an invariant N-x7-R/K-x9 conserved motif residue rich in lysine (K) and arginine (R). The leucine zipper linked to the C-terminus of the basic region contains two sequential heptad repeat peptides, where a leucine (L) is located at the seventh of each peptide. In some cases, the leucine residue is replaced by isoleucine, valine, phenylalanine, or methionine. For CrebZIP proteins, the primary structure of the conserved domain was detected as N-x7-R/K-x9-L-x6-L-x6-L (motif 1), which is consistent with *Arabidopsis* bZIPs [17].

Previous studies showed that there were other special structural domains (e.g., proline-rich, glutamine-rich, and acidic domains) in plant bZIP proteins. These domains may have transcriptional activation function in regulating downstream target gene expressions [37]. For example, two glutamine-rich (~30% Gln) domains adjacent to the C terminal of a bZIP protein encoded by *PERIANTHIA* (*PAN*) in *Arabidopsis* were supposed to act as transcriptional activation domains [38]. A glutamine-rich region in the C-terminal halves of wheat bZIP family members HBP-1b (c38) and HBP-1b (c1), was reported to activate transcription of nuclear genes [39]. STGA1 (soybean TGA1), a member of the TGA (TGACG motif binding factor) subfamily of soybean bZIP TFs, contained a C-terminal glutamine-rich region as a putative transcription activation domain [40]. The conserved motifs shared by *Arabidopsis*, wheat, soybean, and other plants, suggested a similar function for the bZIP proteins. In this study, most *C. reinhardtii* bZIP protein sequences also contained a glutamine-rich region (motif 2), indicating that like higher plants, unicellular microalgae may retain structural domains of important functions during evolution, although the roles of these additional conserved motifs found in CrebZIP proteins are not yet clear.

### 2.3. Analysis of CrebZIP Gene Structure and Their Chromosomal Assignment

**Figure 3:**
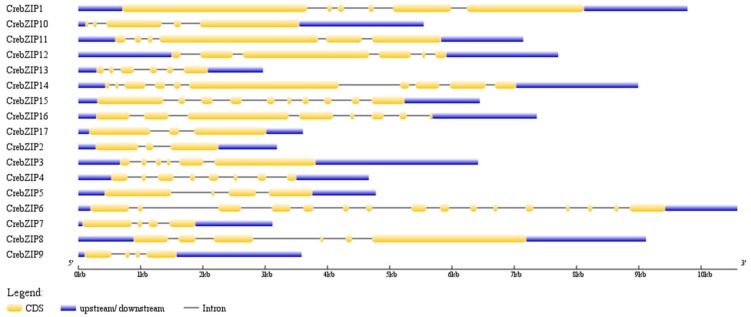
The Exon-intron organization of *CrebZIP* genes. The exons and introns are represented by yellow boxes and horizontal black lines, respectively. Untranslated regions are shown with blue boxes. The length of *CrebZIP* genes are indicated by the horizontal axis (kb).

To further understand the evolutionary relationships among *CrebZIP* genes, GSDS (Gene Structure Display Server) was used to analyze their intron-exon structures. As shown in Figure 3, the number of exons varied from 3 to 16, demonstrating a great divergence among the 17 *CrebZIP* genes. The exon-intron structures of the genes were also highly different even in the same subfamily despite six exons were conserved in the subfamily composed of *CrebZIP8* and *CrebZIP12*. For instance, *CrebZIP6* and *CrebZIP10* grouped as a subfamily, with *CrebZIP6* having 16 exons and *CreZIP10* consisting of 5 exons. For the same subfamily of *CrebZIP9* and *CrebZIP14*, the former had 4 exons while the later contained 10 exons. Such variance in intron-exon structures was also found in *bZIP* genes of rice (*Oryza sativa*), soybean (*Glycine max*), and strawberry (*Fragaria ananassa*) plants. Among the *OsbZIP* genes having introns, the number of introns in open reading frames (ORF) varied from 1–12, 1–18, and 1–20 in rice [41], soybean [42], and strawberry plants [43], respectively. In agreement with these previous findings, this diversity in exon-intron organization indicated that both exon loss and gain occurred during the evolution of the *C. reinhardtii bZIP* gene family.

**Figure 4:**
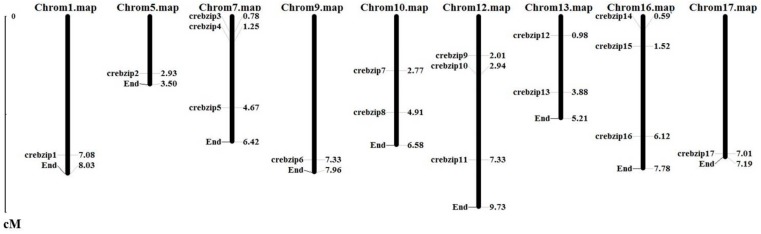
Distribution of *CrebZIP* gene family members on the *C. reinhardtii* chromosomes. The chromosome number is indicated at the top of each chromosome. Values next to the *bZIP* genes indicate the location on the chromosome.

Chromosome assignment of CrebZIP genes depicted by MapInspect software displayed 17 *CrebZIP* genes unevenly distributed on nine chromosomes of *C. reinhardtii*. Three *CrebZIP* genes were located on chromosomes 7, 12, and 16. Chromosomes 10 and 13 both had two *CrebZIP* genes. Only one *CrebZIP* gene was found on chromosomes 1, 5, 9, and 17 (Figure 4). In addition, several *CrebZIP* genes including *CrebZIP1*, *2*, *6*, *13*, and *17*, were distributed near the ends of chromosomes.

### 2.4. Characterization of Cell Growth and Lipid Accumulation in C. reinhardtii Under Salt Stress

**Figure 5:**
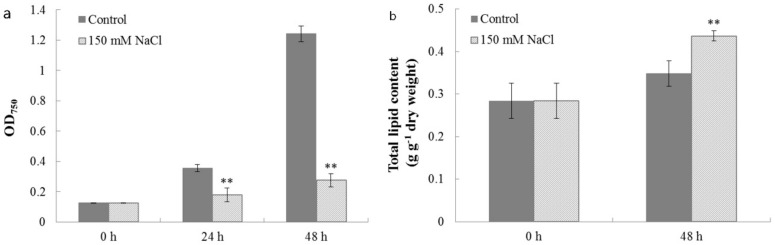
The effects of salt stress on (**a**) cell growth and (**b**) total lipid content of *C. reinhardtii*. Cell samples harvested after 0, 24, and 48 h cultivation from both the salt treatment and the control were used for measurements of cell growth and lipid content. Each value is the mean ± SD of three biological replicates. Asterisks indicate statistical significance (* 0.01 < *p* < 0.05, ** *p* < 0.01) between control and salt-treated cells according to the Tukey’s test.

The effects of salt stress on *C. reinhardtii* growth and lipid accumulation are shown in Figure 5. Salt stress (150 mM NaCl treatment) significantly affected the cell growth, with OD values increasing slowly from 0.127 ± 0.002 to 0.276 ± 0.044 during 48 h cultivation, while the cell growth curve showed a rapid increase in the control with OD values from 0.127 ± 0.002 to 1.242 ± 0.052 (Figure 5a). In contrast, total lipid content in *C. reinhardtii* cells under salt stress significantly increased from 0.284 ± 0.029 to 0.437 ± 0.012 (Figure 5b), whereas lipid content in the control only increased at a small scale from 0.284 ± 0.029 to 0.348 ± 0.022, after 48 h cultivation. Consistent with these findings, Kato et al. observed that salinity stimulated lipid accumulation, but negatively affected biomass production in microalgae [44].

**Figure 6:**
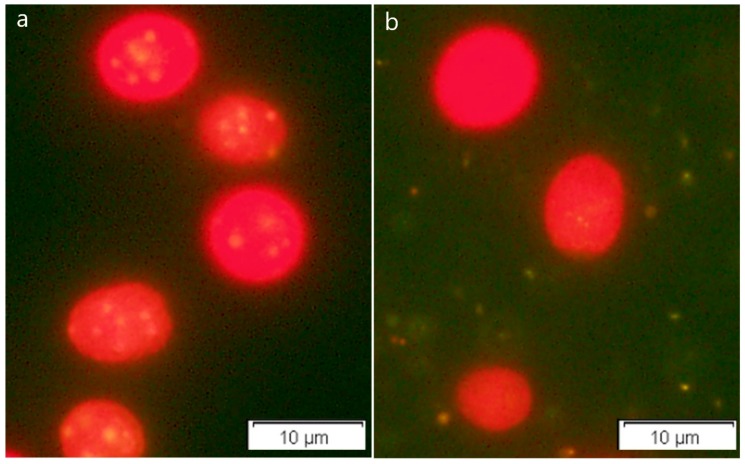
Nile Red staining lipid drops in *C. reinhardtii* under (**a**) salt stress and (**b**) the control treatments. The cell and lipid droplet colors were visualized with red and yellow, respectively, under fluorescent light.

Oil accumulation in *C. reinhardtii* cells under salt stress was also examined by fluorescent microscopy using Nile Red staining (Figure 6). Notably, salt stress resulted in more oil droplets in *C. reinhardtii* cells, which was consistent with the total lipid content (Figure 5b) measured for the stressed cells. Similarly, other reports also showed that salinity stress induced enhancement of total lipid content and neutral lipid fractions within microalgae cells by affecting the fatty acid metabolism [45,46,47]. It is possible that in response to the decrease of cell membrane osmotic pressure and fluidity caused by salt stress, microalgae could employ an adaptive strategy to accumulate neutral lipid TAG, so as to maintain membrane integrity.

### 2.5. Photosynthetic Properties of C. reinhardtii Cells under Salt Stress

**Figure 7:**
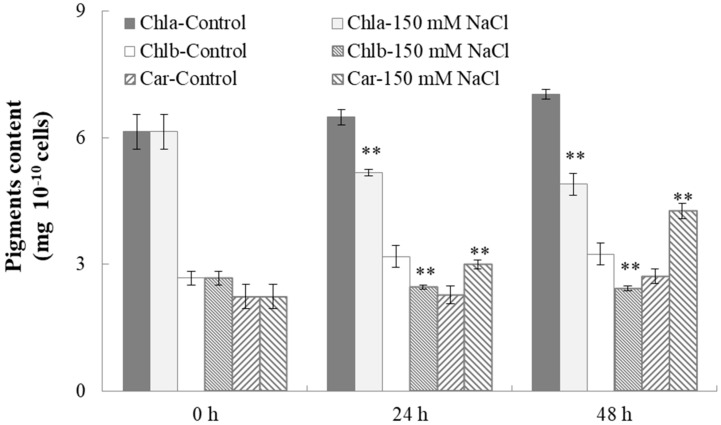
The effect of salt stress on pigment contents of *C. reinhardtii*. Cell samples harvested after 0, 24, and 48 h cultivation from both the salt treatment and the control were used to measure the contents of chlorophyll a, Chlorophyll b, and carotenoid. Each value is the mean ± SD of three biological replicates. Asterisks indicate statistical significance (* 0.01 < *p* < 0.05, ** *p* < 0.01) between control and salt-treated cells according to the Tukey’s test.

To investigate the effects of salt stress on *C. reinhardtii* photosynthesis, the contents of three pigments, Chlorophyll a (Chla), Chlorophyll b (Chlb), and carotenoids (Car), were measured. As shown in Figure 7, salt stress also led to significant changes in pigment content of *C. reinhardtii*. Under salt stress, Chla and Chlb contents in *C. reinhardtii* both decreased from 6.147 ± 0.409 and 2.680 ± 0.161 to 4.903 ± 0.258 and 2.427 ± 0.0.055 mg 10^−10^ cells, respectively, during 48 h cultivation, showing significant difference from the control where Chla and Chlb contents increased during cultivation. Chlorophyll is the main light-harvesting molecule for photosynthetic organisms. The reduction of chlorophyll content indicated weakened photosynthesis in *C. reinhardtii* under salt stress, possibly being the result of decreased synthesis or enhanced degradation of chlorophylls caused by the stress. It is known that salinity caused limitations in photosynthetic electron transport and then photosynthetic rate [48]. Moreover, salinity may stimulate chlorophyllase activity and accelerate chlorophyll degradation [49]. Unlike the chlorophyll case, the carotenoid content in *C. reinhardtii* grown under salinity increased from 2.240 ± 0.295 to 4.263 ± 0.180 mg 10^−10^ cells after 48 h cultivation, in comparison with the control where carotenoid content exhibited no obvious change during the cultivation. Higher carotenoid content under salt stress in *C. reinhardtii* was possibly due to positive adaptation to the stress, which was also observed in microalgae *Botryococcus braunii* by Rao et al. [50]. It was proved that carotenoids could protect the photosynthetic apparatus from photo-oxidative damage [51], providing the protection mechanism for microalgae cells against adverse stresses.

**Figure 8:**
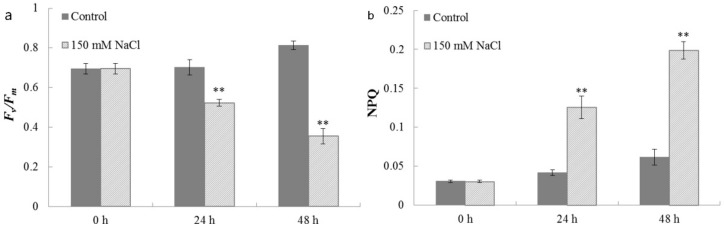
The effects of salt stress on (a) the maximum photo-chemical efficiency (*F_v_*/*F_m_*) and (b) non-photochemical quenching co-efficiency (NPQ) of *C. reinhardtii*. Cell samples harvested after 0, 24, and 48 h cultivation from both the salt treatment and the control, were used for measurements of *F_v_*/*F_m_* and NPQ. Each value is the mean ± SD of three biological replicates. Asterisks indicate statistical significance (* 0.01 < *p* < 0.05, ** *p* < 0.01) between control and salt-treated cells according to the Tukey’s test.

The photosynthetic performance in algae and plants was widely monitored by measuring chlorophyll fluorescence. For example, the *F_v_*/*F_m_* parameter was used for estimating the maximum quantum yield of PSII photochemistry. Non-photochemical quenching (NPQ) was employed to examine changes in the apparent rate constant for excitation decay by heat loss from PSII. NPQ was an essential part of the plant response to stress, as indicated by their slower growth [52]. Decreased *F_v_*/*F_m_* was often observed when plants were exposed to abiotic and biotic stresses [53]. The effect of salt stress on the chlorophyll fluorescence of *C. reinhardtii* is demonstrated in Figure 8. *F_v_*/*F_m_* values decreased from 0.695 ± 0.027 to 0.354 ± 0.038 in salt-treated cells during the 48 h cultivation, while it slightly increased from 0.695 ± 0.027 to 0.812 ± 0.022 in the control (Figure 8a). Meanwhile, NPQ values in cells detected under salt stress and in the control, both increased in this culture period. However, NPQ values under salinity increased more significantly (from 0.030 ± 0.002 to 0.199 ± 0.011) compared to the control (from 0.030 ± 0.002 to 0.061 ± 0.010) (Figure 8b). Analogously, Mou et al. [54] also observed lower *F_v_*/*F_m_* and associated induction of NPQ when *Chlamydomonas* sp. ICE-L was cultured under stress. In addition, they also summarized that the lower *F_v_*/*F_m_* indicated stress conditions, and the higher NPQ showed energy dissipation.

Taken together, salt stress affected various physiological and photosynthetic mechanisms associated with cell growth and development of *C. reinhardtii*. Microalgae cells altered their metabolism to adapt to the adverse environment by reducing biomass production and chlorophyll content, and simultaneously by increasing lipid and carotenoid contents. Moreover, salt stress impeded photosynthesis in *C. reinhardtii*, reflected by decreased *F_v_*/*F_m_* and increased NPQ. Salinity may induce excess production of reactive oxygen species (ROS) which caused cell damage. ROS also participated in regulating the expression of many genes and signal transduction pathways. Under stress, microalgae cells subsequently changed the physiological and photosynthetic status by: (1) Enhancing NPQ to reduce light energy absorption and to accelerate energy dissipation, thus mitigating the damage caused by excess excitation energy. (2) Accumulating lipid at the cost of reduced growth and carbohydrate storage as a response to the oxidative stress [55,56].

### 2.6. The Expression of CrebZIP Genes under Salt Stress

**Figure 9:**
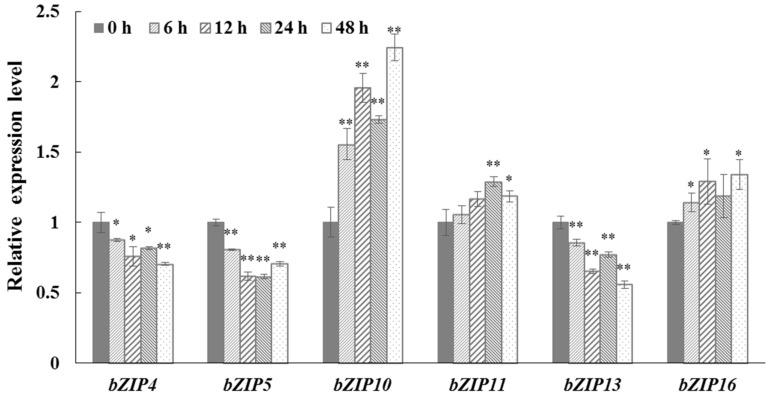
Expression profiles of six *CrebZIP* genes in *C. reinhardtii* under salt stress. Expression of *CrebZIP* genes was determined by qRT-PCR using total RNA from microalgae cells sampled at different time points of salt treatment. The α-tubulin gene was used as the internal reference gene. Each value is the mean ± SD of six biological replicates. Asterisks indicate statistical significance (* 0.01 < *p* < 0.05, ** *p* < 0.01) between control and salt-treated cells according to the Tukey’s test.

To investigate whether any CrebZIP functioned in regulating stress responses and oil accumulation in *C. reinhardtii*, expression analysis of all 17 *CrebZIP* genes following exposure to salt stress by quantitative real-time (qRT)-PCR were performed. Among the 17 *CrebZIP* genes detected, only six genes including *CrebZIP4*, *5*, *10*, *11*, *13*, and *16* showed significant expression changes during salt stress (Figure 9). The rest of 11 *CrebZIP* genes exhibited no obvious expression changes between the salt treatment and the control during 48 h cultivation. The *CrebZIP10*, *11*, and *16* genes were up-regulated, whereas *CrebZIP4*, *5*, and *13* genes were down-regulated under salt stress compared to the control (Figure 9), indicating that these CrebZIP TFs may be involved in the *C. reinhardtii* defense response to salt stress. Similarly, Zhu et al. also observed the obvious expression changes of several *bZIP* genes in tomato (*Solanum lycopersicum*) plants under salt and drought stresses. Furthermore, they concluded that SlbZIP1 mediates the stress tolerance in tomato plants through regulating an ABA-mediated pathway revealed by silencing of the gene and RNA-seq analysis [57]. Consequently, it was speculated that the six CrebZIP TFs may participate in regulation of photosynthesis and oil accumulation in *C. reinhardtii* under stress conditions, based on the association of *CrebZIP*’s expression and physiological phenotypes of the cells. Nevertheless, to verify whether the six CrebZIPs directly mediate the regulation of *C. reinhardtii* cell growth, photosynthesis, and lipid accumulation in response to stresses, mutants of gain-of-function and loss-of-function of these six CrebZIPs should be generated, and correspondingly, phenotypic analysis needs to be conducted on these mutants under stress in future study. The data from this study was the first evidence showing that CrebZIPs may mediate the regulation of stress responses, particularly the oil accumulation under salt stress, although a few CrebZIPs may be involved in regulating N-starving stress responses in *C. reinhardtii* [58].

Although bZIP TFs in higher plants have been intensively explored, the study of bZIP proteins of microalgae have not, limiting the functional analysis of bZIP members in microalgae. The study of bZIP TFs in green plant evolution suggested that the ancestor of green plants possessed four bZIP genes functionally involved in oxidative stress, unfolded protein responses, and light-dependent regulations [15]. Takahashi et al. found that in algae *Vaucheria frigida* and *Fucus distichus*, each of two bZIP proteins, chromoproteins AUREO1 and AUREO2, contained one bZIP domain and one light–oxygen–voltage (LOV)-sensing domain, representing the blue light (BL) receptor. It was hypothesized that because bZIP proteins typically bind DNA by forming heterodimer, AUREO1 and AUREO2 may cooperatively regulate different kinds of BL responses by forming homo- and hetero-dimers [59]. Marie et al. reported that the two bZIP TFs AUREO1a and bZIP10 were likely to be involved in the blue light-dependent transcription of a cyclin gene that regulated the onset of the cell cycle in diatom (*Phaeodactylum tricornutum*) after a period of darkness [60]. Fischer et al. identified a singlet oxygen resistant 1 (SOR1), which was a putative bZIP protein in *C. reinhardtii*, that could stimulate the tolerance of *C. reinhardtii* to high O_2_ formation by activating a reactive electrophile species (RES)-induced defense response, thereby enhancing the tolerance of this organism to photo-oxidative stress [61]. In addition to light-dependent regulation and oxidative stress resistance, bZIP TFs were also proved to participate in TAG accumulation in microalgae. Hu et al. revealed that bZIP family members were dominant (five such TFs) among 11 TFs that were potentially involved in the transcriptional regulation of TAG biosynthesis pathways in *Nannochloropsis* [14]. Consistent with these previous reports, the present study provides new data to show that some bZIP TFs may mediate the regulation of photosynthesis and lipid synthesis in microalgae, especially under stress conditions.

## 3. Materials and Methods

### 3.1. Microalgae Strains and Growth Conditions

The microalgae *C. reinhardtii* was purchased from the Freshwater Algae Culture Collection of the Institute of Hydrobiology, Chinese Academy of Science, China, and maintained in Tris-Acetate-Phosphate (TAP) medium [62]. The culture of *C. reinhardtii* was inoculated in 250 mL flasks under a continuous illumination of 100 μmol m^−2^ s^−1^ and a temperature of 25 ± 1 °C. A certain amount of NaCl was added into the TAP medium to a final concentration of 150 mmol L^−1^ in salt stress treatment, while algal cells were cultivated in normal TAP medium as the control.

### 3.2. Genome-Wide Identification of C. reinhardtii bZIP Gene Family 

The bZIP protein sequence of *C. reinhardtii* was identified and downloaded through the following means: Using HMMMER V3.0 software with the Hidden Markov Model (HMM) PF00170 (from Pfam database, http://pfam.xfam.org/) of bZIP domain, from the Phytozome database (http://phytozome.jgi.doe.gov/pz/portal.html) and Plant Transcription Factor Database (PlnTFDB) with the keyword of bZIP. The screening results of the three means were merged, followed by manually removing the redundant or repetitive sequences. 

The prediction of the bZIP structure domain was carried out within the amino acid sequence of the selected candidate bZIP protein family member in *C. reinhardtii* with the help of the Conserved Domain Database (CDD, http://www.ncbi.nlm.nih.gov/Structure/cdd/wrpsb.cgi) in the National Center for Biotechnology Information (NCBI, http://www.ncbi.nlm.nih.gov/), and Simple Modular Architecture Research Tool software (SMART, http://smart.embl-heidelberg.de). The candidate genes without bZIP structure domain were removed. The physicochemical properties of the bZIP protein of *C. reinhardtii*, including hydropathicity, molecular mass, instable index, and so forth, were predicted via ProtParam software from the Expasy database (http://web.expasy.org/protparam/). 

### 3.3. Motif Recognition and Phylogenetic Analysis of C. reinhardtii bZIP Gene Family

Motifs of the selected genes were analyzed using the MEME suite (http://meme-suite.org/tools/meme). Multiple alignments of bZIP protein sequences were performed by ClustalW software and the phylogenetic tree was constructed with MEGA 7.0 software (https://www.megasoftware.net/home). 

### 3.4. bZIP Protein Secondary Structure Prediction

The secondary structure of bZIP proteins in *C. reinhardtii* was predicted using the PBIL LYON-GERLAND database (https://npsa-prabi.ibcp.fr/cgi-bin/npsa_automat.pl?page=/NPSA/npsa_hnn.html). 

### 3.5. bZIP Gene Structure and Chromosomal Assignment Analysis

The *bZIP* gene structure diagram was constructed by Gene Structure Display Server (GSDS) 2.0 software (http://gsds.cbi.pku.edu.cn/), and then quantitative analysis of introns and exons followed. The chromosome map of *CrebZIP* gene family members was depicted by MapInspect software according to the *CrebZIP* genes location on chromosomes of *C. reinhardtii*.

### 3.6. Measure of Microalgae Biomass Concentration 

The biomass concentration of *C. reinhardtii* was indicated by optical cell density, which was measured with a UV-Visible spectrophotometer (UV-1200, Shanghai Jingke Instrument Co., Ltd., Shanghai, China) at 750 nm (OD_750_). When necessary, the sample was diluted to give an absorbance in the range of 0.1–1.0. The experiments were conducted in triplicate.

### 3.7. Analysis of Total Lipid Content in Microalgae Cells

The total lipid content was determined by gravimetric analysis according to the method of Chen et al. [63]. The microalgae cells were collected by centrifugation and then lyophilized. About 50 mg of lyophilized microalgae sample was triturated in a mortar, and then the cell disruption was added into 7.5 mL chloroform/methanol (1:2, *v*/*v*) mixture. The mixture was placed at 37 °C overnight and then centrifuged to collect the supernatant. Residual biomass was extracted at least once more. All the supernatants were combined, and added into a chloroform and 1% sodium chloride solution to a final volume ratio of 1:1:0.9 (chloroform/methanol/water). The new mixture was centrifuged afterwards, and the subnatant was transferred to a pre-weighted vitreous vial. The sample solution was dried to constant weight at 60 °C under nitrogen flow. Finally, the total lipid content was obtained as a percentage of the dry weight (DW) of the microalgae. The experiments were conducted in triplicate.

### 3.8. Nile Red Staining and Microscopy for Assessment of Oil Accumulation in Microalgae Cells

Lipid droplets were visualized by fluorescent microscopy using Nile Red staining [64]. Microalgae cells under salt stress and control conditions were collected after 48 h cultivation by centrifugation. The cells pellets were washed with physiological saline solution three times. After the collected cells were re-suspended in the same solution, 10 μL Nile Red stain (0.1 g L^−1^ in acetone) and 200 μL dimethyl sulfoxide were added into an 800 μL microalgae suspension, with final concentration at about 1 × 10^6^ cells mL^−1^. The stained cells were incubated in the dark for 20 min at room temperature, and then immediately observed by fluorescent microscopy.

### 3.9 Determination of the Pigment Contents in Microalgae Cells

Contents of microalgae pigments, including chlorophyll (a and b) and carotenoid, were determined according to the methods described by Wellburn [65]. A certain amount of microalgae culture was centrifuged to collect cells. The cell pellets were mixed with methanol at 60 °C for 12 h in darkness, until the microalgae cells whitened completely. The mixture was centrifuged and supernatant extraction was collected. The optical densities of the extraction were measured with a spectrophotometer (UV-1601, Beijing Beifen-Ruili Aanlytical Instrument Co., Ltd., Beijing, China) at 666, 653, and 470 nm. The concentrations of chlorophyll a (Chla), chlorophyll b (Chlb), and carotenoid (Car) of the extraction were calculated as follows (mg L^−1^):

Chla = 15.65 × OD_666_ − 7.34 × OD_653_ (1)

Chlb = 27.05 × OD_653_ − 11.21 × OD_666_ (2)

Car = (1000 × OD_470_ − 2.86 × Chla – 129.2 × Chlb)/221 (3)

The cell density of the microalgae culture was obtained using cytometry with a hemacytometer. The experiments were conducted in triplicate.

### 3.10 Chlorophyll Fluorescence Measurements

The chlorophyll fluorescence was measured using an Imaging-PAM (Pulse Amplitude Modulation) fluorescence monitor (Walz, Effeltrich, Germany) according to the method described by Mou et al. [54]. Samples were dark adapted for 15 min before the fluorescence measurement. Then the dark-level fluorescence yield (*F*_0_), the maximum fluorescence yield (*F_m_*), and the maximum light-adapted fluorescence yield (*F*’*_m_*) were measured. 

The maximum quantum yield of PSII was calculated as:

*F_v_*/*F_m_* = (*F_m_* − *F*_0_)/*F_m_* (4)

The non-photochemical quenching (NPQ) was calculated as:

NPQ = (*F_m_* − *F’_m_*)/*F’_m_* (5)

The experiments were conducted in triplicate.

### 3.11 RNA Isolation and Quantitative Real-Time (qRT)-PCR Analysis

*CrebZIP* genes expression under salt stress were analyzed by qRT-PCR. Total RNA of *C. reinhardtii* cells sampled after 0, 6, 12, 24, and 48 h cultivation under salt stress were extracted. RNA from each sample was used to synthesize the cDNAs with the cDNA Synthesis Kit (TaKaRa, Kusatsu, Japan). The primers for the 17 *CrebZIP* genes and one reference gene (α-tubulin) were designed using Primer Premier 5.0 software. Sequences of all the primers used in this study are shown in Table 3. The qRT-PCR was performed in an ABI 7500 qRT-PCR system (Applied Biosystems, Foster City, CA, USA) with following reaction conditions: 95 °C for 3 min followed by 40 cycles of 95 °C for 15 s, 60 °C for 30 s, and 72 °C for 30 s. The experiments were repeated six times using independent RNA samples.

## 4. Conclusions

The first genome-wide analysis of the bZIP TF family members in *C. reinhardtii* was performed in this study, with a total of 17 CrebZIP proteins identified. Detailed information was obtained for their evolutionary relationship, exon-intron organization and chromosome assignment, protein structural features, and conserved motifs. Moreover, expression profiling of *CrebZIP* genes by qRT-PCR indicated that six CrebZIPs might be involved in stress response and lipid accumulation in microalgae cells. Salt stress led to the reduction in biomass production, chlorophyll content, and *F_v_/F_m_*, but the enhancement in NPQ, carotenoid content, and oil accumulation in *C. reinhardtii*. Collectively, integration of findings in the present study provided new data to indicate that some CrebZIP TFs could play important roles in mediating regulation of cell growth, photosynthesis, and oil accumulation in microalgae, particularly under stress conditions. These *CrebZIP* genes could be utilized to further functionally characterize them, laying the foundation for elucidating their specific regulatory mechanisms and ultimately applying them in genetic improvement programs.

## Figures and Tables

**Table 1:** Physicochemical properties of *CrebZIP* gene coding proteins.

Gene Number	NCBI Accession Number	Phytozome Identifier	Chromosome Localization (bp)	Protein Length (aa)	Molecular Weight (Da)	Theoretical pI	Hydrophility	Instability Index
*CrebZIP1*	PNW88934.1	Cre01.g051174	Chr.1: 7078516–7088297	2018	198,080.64	6.00	−0.422	68.82
*CrebZIP2*	PNW83651.1	Cre05.g238250	Chr.5: 2933060–2936249	524	53,624.16	5.47	−0.045	58.98
*CrebZIP3*	PNW80451.1	Cre07.g318050	Chr.7: 782941–789359	802	82,929.15	6.16	−0.406	59.38
*CrebZIP4*	PNW80535.1	Cre07.g321550	Chr.7: 1255642–1260306	393	41,429.96	5.48	−0.510	44.72
*CrebZIP5*	PNW81157.1	Cre07.g344668	Chr.7: 4675427–4680202	750	72,176.41	6.18	−0.046	65.59
*CrebZIP6*	PNW79382.1	Cre09.g413050	Chr.9: 7331940–7342521	1053	106,414.13	6.42	−0.401	54.24
*CrebZIP7*	PNW77489.1	Cre10.g438850	Chr.10: 2769177–2772294	485	49,874.20	6.64	−0.678	58.82
*CrebZIP8*	PNW77864.1	Cre10.g454850	Chr.10: 4910181–4919296	1363	128,662.31	8.75	−0.249	64.61
*CrebZIP9*	PNW74780.1	Cre12.g510200	Chr.12: 2010086–2013670	353	35, 933.29	6.36	−0. 398	58.58
*CrebZIP10*	PNW74984.1	Cre12.g501600	Chr.12: 2939321–2944866	902	89,920.31	6.25	−0.274	56.84
*CrebZIP11*	PNW75863.1	Cre12.g557300	Chr.12: 7330581–7337724	1526	154,908.24	9.40	−0.719	65.07
*CrebZIP12*	PNW73681.1	Cre13.g568350	Chr.13: 981891–989596	1150	114,710.75	9.55	−0.563	68.90
*CrebZIP13*	XP_001693067.1	Cre13.g590350	Chr.13: 3885273–3888238	334	34,514.92	4.96	−0.172	58.83
*CrebZIP14*	PNW71231.1	Cre16.g692250	Chr.16: 591049–600037	1525	153,084.80	5.30	−0.558	72.50
*CrebZIP15*	PNW71414.1	Cre16.g653300	Chr.16: 1524635–1531081	867	90,393.04	6.11	−0.374	55.17
*CrebZIP16*	PNW72253.1	Cre16.g675700	Chr.16: 6123854–6131215	1172	115,285.37	6.48	−0.512	46.46
*CrebZIP17*	PNW71098.1	Cre17.g746547	Chr.17: 7009739–7013345	767	75,438.72	6.01	−0.379	55.72

**Table 2:** Secondary structure of CrebZIP proteins.

Gene Number	Alpha Helix (%)	Extended Strand (%)	Beta Bridge (%)	Random Coil (%)
*CrebZIP1*	24.58	2.68	0	72.75
*CrebZIP2*	53.24	1.53	0	45.23
*CrebZIP3*	36.41	8.73	0	54.86
*CrebZIP4*	45.29	1.53	0	53.18
*CrebZIP5*	41.60	0.80	0	57.60
*CrebZIP6*	43.49	2.94	0	53.56
*CrebZIP7*	45.80	0.42	0	53.78
*CrebZIP8*	30.01	3.67	0	66.32
*CrebZIP9*	36.54	8.22	0	55.24
*CrebZIP10*	38.03	3.77	0	58.20
*CrebZIP11*	26.34	4.59	0	69.07
*CrebZIP12*	39.57	3.22	0	57.22
*CrebZIP13*	50.30	3.59	0	46.11
*CrebZIP14*	39.08	1.70	0	59.21
*CrebZIP15*	46.25	1.85	0	51.90
*CrebZIP16*	33.19	1.88	0	64.93
*CrebZIP17*	27.38	3.00	0	69.62

**Table 3:** Primer information of *CrebZIP* and reference genes for quantitative real-time (qRT)-PCR.

Gene Number	Forward Primer (5′-3′)	Reverse Primer (5′-3′)
*CrebZIP1*	CGGTCGATGACGCTAAGGC	TGGTCGGTGTCGGAGGAGT
*CrebZIP2*	GAGCGCAAGAAGCAGTACGTGACCT	GATGTTCCGCAGTGCCTCGTTCT
*CrebZIP3*	ATGCACCAAACGCCAAATCG	ATCTGCTGTAGGTCCGCCAGGGTA
*CrebZIP4*	GACAGCGGAGACTCAGACAT	CCCTTCTTACGCTGCCTGTA
*CrebZIP5*	CCGTCATCCATGCACCTACT	GCTGAAGATCAAGACCGCTG
*CrebZIP6*	CCCGTTTCCAGCCCATCAGAC	TGCAAGGCCGAGGGTGTTGATGAC
*CrebZIP7*	TGGGAGGCTCTGGCTTTGG	TGGCTGCTGCTGCTGCTGATG
*CrebZIP8*	GCAAAGGCAAGGGCAAGG	CGCAGGAGTTCTCAGCCGATT
*CrebZIP9*	GCGCTTCTTCCTCGCTACCA	CGCAAGCCCTTGTGCTGTTA
*CrebZIP10*	CCCTGACGTCCACCTTAACT	TCCCTCAGACAGCAACTCAG
*CrebZIP11*	ACGGACGTATGACATGAGCA	ACTGCTGGAACTGGTGGAA
*CrebZIP12*	CACAGCGACCGCAGCCATAA	GCCCAGCTTGTCCGAGAAGGA
*CrebZIP13*	GAGCTGCCTTCGACAAGC	CTGTGCTAGGCGATTCAGC
*CrebZIP14*	GATGGCGGGCTTATGTCGG	CAAGGCGTCCACGTTGTG
*CrebZIP15*	TGACTCATCCACGCACTTCCTC	TGATGTTGCACCAGCCCTGA
*CrebZIP16*	GGTTTCCTTCCCTACCCA	ACGGCACGGTTGTCAGCA
*CrebZIP17*	AAGCGCATTGTTGACGGAG	CGCAGCAGGTCTAATAAGTCG
Creα-tubulin	CTCGCTTCGCTTTGACGGTG	CGTGGTACGCCTTCTCGGC

## Supplementary Materials

Supplementary materials can be found at http://www.mdpi.com/1422-0067/19/9/2800/s1.
