# Supplementary material for: Analysis of bZIP Transcription Factor Family and Their Expressions under Salt Stress in Chlamydomonas reinhardtii
Source: Int J Mol Sci. 2018 Sep 17;19(9):2800. doi: 10.3390/ijms19092800 (PMC6164503; doi:10.3390/ijms19092800)
Supplement: Supplementary file 1 [file ijms-19-02800-s001.pdf]

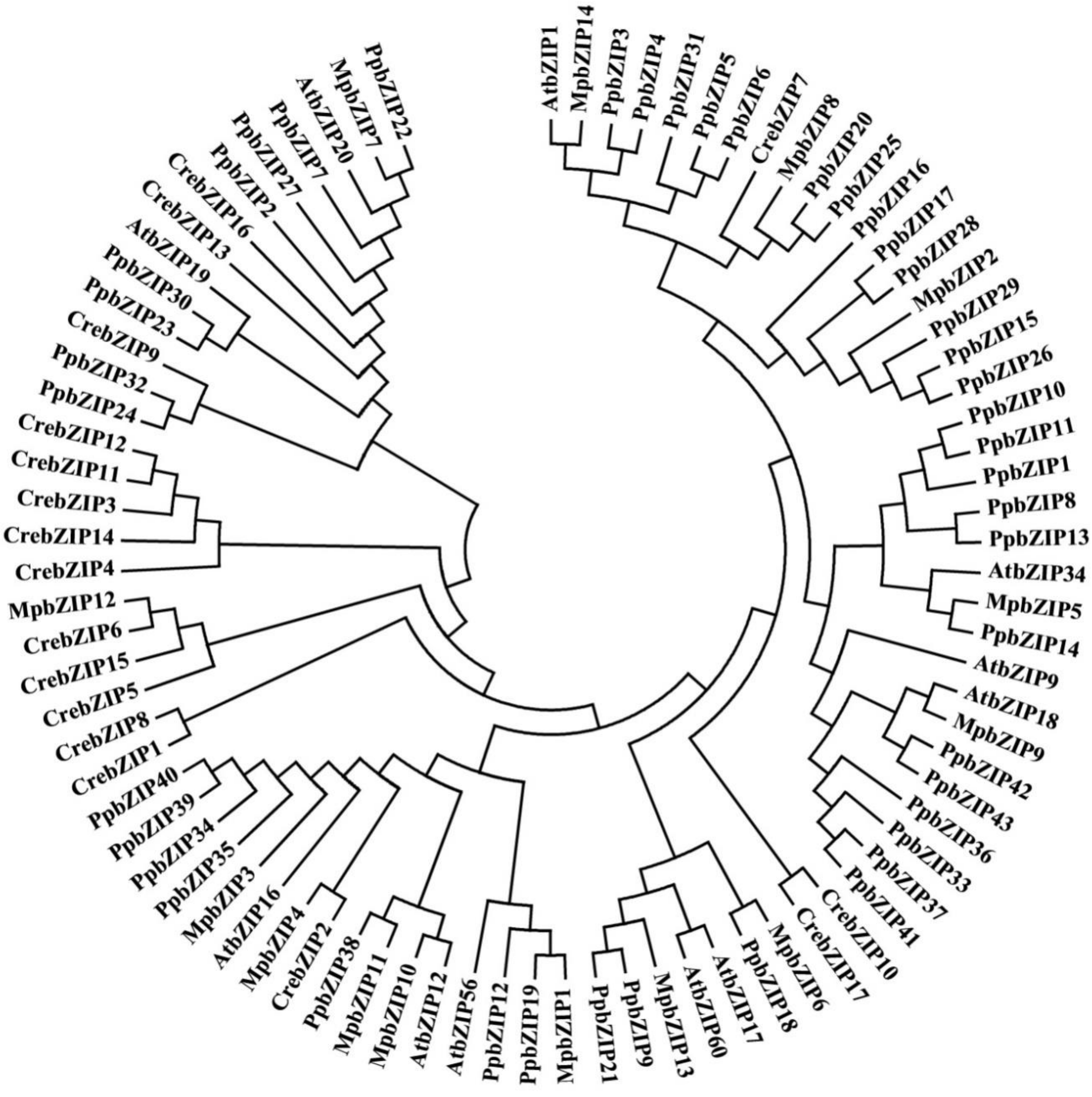

Figure S1. Phylogenetic analysis of *C. reinhardtii*, *Arabidopsis*, *P. patens*, and *M. polymorpha* bZIP proteins. ClustalW was employed to align the protein sequences of 17 CrebZIPs, 43 PpbZIPs, 14 MpbZIPs, and 11AtbZIPs, representing subgroups A to I, S, and U, respectively, in *Arabidopsis*. The phylogenetic tree was constructed using the neighbor-joining (NJ) method with MEGA 7.0 software.

Table S1. Details of *PpZIP* and *MpbZIP* genes identified from *P. patens* and *M. polymorpha*.

Gene number	NCBI accession Number	Phytozome identifier	Gene number	NCBI accession Number	Phytozome identifier
PpbZIP1	XP_024387430.1	Pp3c1_7300	*PpbZIP30*	XP_024400833.1	Pp3c17_11650
PpbZIP2	XP_024379754.1	Pp3c1_21760	*PpbZIP31*	XP_024399812.1	Pp3c17_21430
PpbZIP3	PNR63101.1	Pp3c1_33070	*PpbZIP32*	XP_024401501.1	Pp3c17_21620
PpbZIP4	XP_024377704.1	Pp3c1_33080	*PpbZIP33*	XP_024403159.1	Pp3c18_14260
PpbZIP5	XP_024376758.1	Pp3c1_38440	*PpbZIP34*	XP_024402784.1	Pp3c18_16260
PpbZIP6	XP_024399762.1	Pp3c2_4600	*PpbZIP35*	XP_024403163.1	Pp3c18_19240
PpbZIP7	XP_024397994.1	Pp3c2_16080	*PpbZIP36*	XP_024356505.1	Pp3c19_15700
PpbZIP8	XP_024368462.1	Pp3c2_26970	*PpbZIP37*	PNR34360.1	Pp3c19_15730
PpbZIP9	XP_024371307.1	Pp3c3_16820	*PpbZIP38*	PNR32900.1	Pp3c20_7290
PpbZIP10	XP_024376312.1	Pp3c5_20330	*PpbZIP39*	XP_024359043.1	Pp3c21_5710
PpbZIP11	XP_024378642.1	Pp3c6_7520	*PpbZIP40*	XP_024359043.1	Pp3c21_5770
PpbZIP12	XP_024381386.1	Pp3c7_11360	*PpbZIP41*	XP_024360862.1	Pp3c22_13770
CrebZIP13	XP_024379985.1	Pp3c7_23340	*PpbZIP42*	XP_024360145.1	Pp3c22_13810
PpbZIP14	XP_024383474.1	Pp3c8_11470	*PpbZIP43*	XP_024361112.1	Pp3c22_13840
PpbZIP15	XP_024384443.1	Pp3c9_2770	*MpbZIP1*	PTQ49943.1	Mapoly0001s0021
PpbZIP16	XP_024385346.1	Pp3c9_6339	*MpbZIP2*	PTQ46245.1	Mapoly0012s0172
PpbZIP17	XP_024385345.1	Pp3c9_6350	*MpbZIP3*	PTQ45180.1	Mapoly0015s0005
PpbZIP18	XP_024388291.1	Pp3c11_3960	*MpbZIP4*	PTQ45041.1	Mapoly0016s0098
PpbZIP19	PNR45393.1	Pp3c11_17710	*MpbZIP5*	PTQ44602.1	Mapoly0019s0040
PpbZIP20	XP_024392804.1	Pp3c13_15010	*MpbZIP6*	PTQ43992.1	Mapoly0022s0095
PpbZIP21	XP_024392785.1	Pp3c13_15030	*MpbZIP7*	PTQ43153.1	Mapoly0026s0039
PpbZIP22	XP_024394712.1	Pp3c14_4880	*MpbZIP8*	OAE35970.1	Mapoly0034s0126
PpbZIP23	XP_024394864.1	Pp3c14_6250	*MpbZIP9*	OAE30479.1	Mapoly0046s0102
PpbZIP24	XP_024394677.1	Pp3c14_19470	*MpbZIP10*	OAE26867.1	Mapoly0069s0009
PpbZIP25	XP_024396092.1	Pp3c15_5820	*MpbZIP11*	PTQ35305.1	Mapoly0072s0050
PpbZIP26	XP_024396641.1	Pp3c15_7040	*MpbZIP12*	PTQ30588.1	Mapoly0122s0020
PpbZIP27	PNR39364.1	Pp3c15_11920	*MpbZIP13*	PTQ30075.1	Mapoly0130s0030
PpbZIP28	XP_024397438.1	Pp3c15_18260	*MpbZIP14*	PTQ26645.1	Mapoly0737s0001
PpbZIP29	XP_024397437.1	Pp3c15_18270			
